# Evaluation of Phytase Impact on *In Vitro* Protein and Phosphorus Bioaccessibility of Two Lupin Species for Rainbow Trout (*Oncorhynchus mykiss*)

**DOI:** 10.1155/2024/2697729

**Published:** 2024-01-03

**Authors:** Rosendo L. Azcuy, Matías E. Casaretto, Lorenzo Márquez, Adrián J. Hernández, Gabriel A. Morales

**Affiliations:** ^1^Facultad de Agronomía, Departamento de Producción Animal, Cátedra de Acuicultura, Universidad de Buenos Aires, Buenos Aires, Argentina; ^2^Centro de Investigación, Innovación y Creación UCT, Facultad de Recursos Naturales, Universidad Católica de Temuco, Temuco, Chile; ^3^Núcleo de Investigación en Producción Alimentaria, Departamento de Ciencias Agropecuarias y Acuícolas, Facultad de Recursos Naturales, Universidad Católica de Temuco, Temuco, Chile; ^4^Instituto de Investigaciones en Producción Animal, Consejo Nacional de Investigaciones Científicas y Técnicas INPA–CONICET, Buenos Aires, Argentina

## Abstract

Legumes are an important source of protein, lipids, and other essential nutrients. As the demand for protein and lipids continues to surge on a global scale, there is a growing interest in incorporating legumes into aquafeeds. This shift is driven not only by the escalating growth of the aquaculture sector in recent years but also by the imperative to diminish the dependency on traditional resources like fishmeal (FM) and fish oil. Amongst legumes, different lupin species had been identified as a potential protein source to partially reduce the inclusion of FM in countries such as Australia, Chile, and the European Union. A comprehensive evaluation of their nutritional profiles, overall characteristics, and potential antinutritional factors is essential for informed utilization and the implementation of nutritional enhancement strategies. In pursuit of this goal, an *in vitro* gastrointestinal simulation system was devised to replicate the digestive conditions of rainbow trout (*Oncorhynchus mykiss*). The study focused on determining the bioaccessibility of protein and phosphorus within two sweet lupin varieties (alkaloids < 0.05) with high (*Lupinus mutabilis*) and low (*Lupinus angustifolius*) native phytic acid content evaluated as whole (W) or dehulled (D) seeds meals and the effect of a single dose of phytase (2,500 FTU/kg DM). Additionally, regular soybean meal (SBM) served as reference (10 treatments with 3 replicates). A 2,500 FTU/kg DM phytase dose increased the levels of PO_4_^−3^ released throughout the intestinal phase by 122.6% for *L. mutatabilis* W, 116.3% for *L. mutatabilis* D, 65.2% for *L. angustifolius* W, 59.0% for *L. an*gustifolius D, and 91.8% for SBM compared to controls without phytase. The bioaccessibility of amino acids in varieties treated with phytase increased with respect to the control without phytase. *L. mutabilis* was found to be a potentially viable alternative as a good quality protein source for the manufacture of environmentally friendly aquafeeds.

## 1. Introduction

Given the substantial impact of feed costs on overall production expenses in carnivorous fish farming, as highlighted by Hasan [1], the exploration of alternative options to replace fishmeal (FM) as the primary protein source has become imperative [2, 3]. While the aquaculture industry continues to rely on FM, its limited availability and high costs pose challenges for sustainable production [4–6]. In response to these challenges, there has been a noticeable surge in the incorporation of plant ingredients in aquafeeds, particularly in the feeding of cultured salmonids over the past years [7–10].

One such promising alternative is lupin, a member of the *Lupinus* genus widely distributed globally, with several species cultivated for human or animal consumption. White lupin (*Lupinus albus*) seeds, in general, contain around 36% protein and 9%–16% oil, similar to that found in soy and peanuts. White lupin is a winter-spring crop characterized by a pivoting root that can release otherwise blocked phosphorus (P) in the soil, making it a valuable asset for agriculture. Additionally, as a legume, it possesses the ability to fix atmospheric nitrogen. These attributes make it an excellent choice for incorporation into agricultural rotations, serving as green manure or as a nutrient source for animal or human nutrition [11]. Moreover, certain lupin varieties exhibit the potential to partially replace FM in aquaculture feed formulations [12, 13]. These lupin varieties not only provide substantial protein content but also contribute with P and a variety of essential minerals, as detailed in Table 1.

However, the utilization of plant-based ingredients in feed formulations for carnivorous species is conditioned by various factors, primarily antinutritional factors, like protease inhibitors, phytic acid, saponins, tannins, cyanides, oxalates, gossypol, nonstarch polysaccharides, phytoestrogens, mimosines, which directly or indirectly condition the *in vivo* performance of the amount of feed supplied to the fish [14, 15]. Among these factors are certain compounds that are not harmful in themselves, but their interactions with other elements can result in a negative effect on growth and, therefore, on production. Some of these factors, like protease inhibitors, can be neutralized during feed processing [16], while others, such as phytic acid, remain stable. Phytin, a salt of IP6, is a form of P storage that encompasses about 80% of total P in legumes and cereals [17], and the P element they contain is considered unavailable to fish [18].

In the early stages of industrial aquaculture, approximately 20% of the P present in most fish diets was retained, with the remaining 80% being released into the environment [19–21]. However, current diets have significantly improved, with fish now retaining around 40% of the dietary phosphorus [22]. As phosphorus is recognized as a limiting factor in the productivity of aquatic ecosystems [23, 24], the efficient utilization of dietary P is of great importance in minimizing discharge levels into the aquatic environment. Enhancing phosphorus bioavailability and retention not only contributes to the sustainability of aquaculture but also plays a crucial role in mitigating environmental impact.

The IP6 forms stable structures due to a high-phosphate content and its negative charges, over a wide pH range. IP6 is a chelator of cations such as Ca^2+^ and Mg^2+^, and therefore, the IP6-cation-protein or IP6-cation-AA complexes can occur, making the protein inaccessible to the action of the proteases [25]. The low digestibility of IP6 and its secondary and tertiary complexes cause the highest proportion of P-IP6 (and proteins and cations captured in these complexes) to be excreted into the environment, causing pollution by algal blooms [5]. Likewise, the chelating power of IP6 can facilitate the capture of cations such as calcium, magnesium, zinc, copper, iron, and potassium to form insoluble salts, affecting the absorption and digestion of these essential minerals [26–28]. Finally, IP6 can also interact with proteins, amino acids (AAs), carbohydrates, and lipids, reducing the digestibility of these nutrients [28, 29].

Phytase, myo-inositol (1, 2, 3, 4, 5, 6) hexakis phosphohydrolase, is a group of enzymes widely distributed in nature that catalyzes the sequential release of phosphate from IP6. These enzymes can be of bacterial, fungal, vegetable origin or from the microbiota present in the digestive tract of some animals. They can also be distinguished as thermophilic by their ability to resist temperature variation and in acidic or alkaline, depending on their dephosphorylase activity at different pH ranges [30].

The use of the enzyme phytase as an additive in the formulation of the feed could contribute to greater use of the protein and P contained in the form of IP6. It is well-established that incorporating phytase in feed formulations with a high proportion of plant-based protein and minimal added inorganic phosphorus yields positive effects on phosphorus utilization [31]. The activity of phytase at the gastric and intestinal phases releases bioaccessible P in the form of orthophosphates (PO_4_^3−^). Consequently, those proteins retained by IP6 in complexes with other positively charged elements (Ca, Mg, Mn, Fe) are also released, leaving them accessible to the protease hydrolysis in both gastric and intestinal phases.

To evaluate the nutritional attributes of lupin, two sweet lupin varieties from Semillas Baer (Chile) were selected. One variety, *Lupinus mutabilis* E44, is characterized by a high phytic acid content, while the other, *Lupinus angustifolius* LILA, has a comparatively low phytic acid content. These lupin varieties underwent two levels of processing, with one involving the removal of the seed hull (-D-) and the other without this modification (-W-) while also testing a single dose of phytase enzyme as an additive. The carried-out assay seeks to advance our knowledge of *L. mutabilis* and *L. angustifolius* in terms of nutrient quality: soluble protein, soluble phosphorus, and AAs. It also explores the effect of phytase, as an enzymatic additive, on the release of the same nutrients from the two *Lupinus* matrixes through an *in vitro* digestion system.

## 2. Materials and Methods

### 2.1. Chemical Composition of the Raw Materials

**Figure 1:**
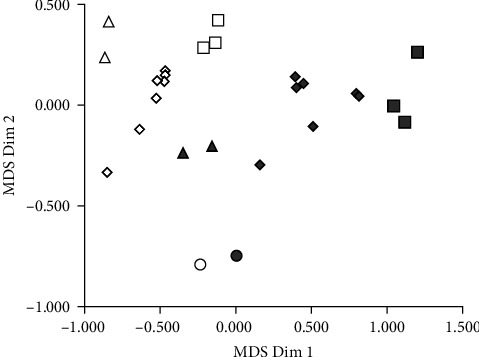
MDS plot of different species and varieties of Lupinus and soybean meal according to their composition of macronutrients and phytate-P. Each symbol represents a different variety. Diamonds: *Lupinus albus*. Squares: *Lupinus angustifolius*. Triangles: *Lupinus mutabilis*. Circles: SBM. Open symbols: dehulled seeds. Gray symbols: whole seeds.

Similarities between the 26 plant meals (12 varieties from 3 species of *Lupinus* plus soybean, all of them analyzed as whole seeds and after dehulling) were evaluated by a multidimensional scaling (MDS) procedure based on Euclidean distances calculated from nutritional compositions. The selected compositional variables were crude protein (CP), total lipids (TL), crude fiber (CF), ash, phytate-P, and ICP-P. The percentage of nitrogen-free extract (NFE) was not included because it was calculated as a difference and depends on the values of the other variables. Each plant meal was assigned the coordinates of the two first dimensions extracted by the MDS procedure and was subsequently represented in a bidimensional MDS plot (Figure 1).

Lupin species were selected according to their total nitrogen (N) and phosphorus (P) content present in the form of phytic acid or phytate and for their total soluble P content according to laboratory proximal profiles. The selected species were *L. mutabilis* (E44) and *L. angustifolius* (Lila) in two presentations (with hull—whole-W or dehulled-D—for lupins), and soybean meal (SBM), included as a known commercial reference. The proximate composition (CP, crude lipid, total fiber, ash, and moisture) of samples was determined according to AOAC [32]. Dry matter was calculated by gravimetric analysis following oven-drying at 105°C for 24 hr. Protein levels were calculated from the determination of total nitrogen by Kjeldahl digestion (based on *N* × 6.25). Fat content was determined gravimetrically following extraction of the lipids with solvent (Soxhlet). Ash content was determined gravimetrically following loss of mass after combustion of samples in a muffle furnace at 550°C for 3 hr. Fiber content was calculated by gravimetric analysis following oven-drying at 105°C for 24 hr and acid and alkali digestion with sulfuric acid and sodium hydroxide respectively. NFE content was determined by difference (100 − % CP + % lipids + % ash + % CF). Phosphorus concentrations were determined according to Engelen et al. [33] using a molybdovanadate reagent and spectro-photochemical measurement at 415 nm (Lambda 25 UV/VIS Spectrometer, PerkinElmer, USA). Phytic acid determinations were analyzed according to Latta and Eskin [34] using a rapid method described for the colorimetric determination of 1.5–15 *µ*g phytate phosphorus in concentrations as low as 3 *µ*g/ml in extracts of cereal grains and cereal products. The phytic acid is precipitated with an acidic iron (III)—solution of known iron content. The decrease of iron in the supernatant is a measure for the phytic acid content. Digestion of the samples for minerals analyses was conducted with nitric acid using the START-D Microwave Digestion System (Milestone Ltd., Bergamo, Italy). The measurements of minerals were performed using the PerkinElmer Optima 5300 DV ICP-OES instrument (Shelton, CT, USA), which was equipped with WinLab32® software for simultaneous measurement of all analyte wavelengths of interest.

### 2.2. Source of Enzyme Extract

A group of adult rainbow trout *Oncorhynchus mykiss* individuals with average weight 346.0 ± 15.6 g (*n* = 10) was reared at 17 ± 1°C and 12 : 12 photoperiod and fed daily to 4.0% body weight with a CP balanced feed during more than 20 days. This group of fish, acclimated to a known diet for 2 weeks, were starved for 48 hr, anesthetized with benzocaine (25 mg/l), weighted, sacrificed, and the stomachs and guts were dissected, and cover fat cleaned. Samples of both tissues were separately homogenized using a mixer (IKA® ULTRA-TURRAX®) in cold distilled water (1 : 5 w/v; 4°C) and then centrifuged at 17,000 × *g* for 15 min. After the extraction of the superficial lipid layer, the supernatants were pooled and stored at –20°C, being further utilized for enzymology assays. All procedures performed in the present study involving animals were in accordance with the ethical standards of the Faculty of Agronomy, University of Buenos Aires.

### 2.3. Protease Activities from Enzyme Extract and Exogenous Phytase Activity

Total acid protease activity was assayed using 10 *μ*l of gastric enzyme extract and 1.0 ml of a substrate solution of 0.5% hemoglobin in 100 mM Gly-HCl buffer pH 2 according to Anson [35]. The mixtures were incubated for 20 min at 17°C, and the reaction was stopped by adding 0.5 ml of 20% trichloroacetic acid (TCA). Blank tubes were prepared similarly, except from the enzyme extract that was added after the TCA solution. The absorbance of the TCA-soluble peptides was measured at 280 nm. One unit of enzyme activity was defined as 1 *μ*g of tyrosine released/min, using the molar extinction coefficient for tyrosine 1.280 M^−1^/cm.

Total alkaline protease activity was determined according to Walter [36]. In this case, the substrate consisted of a 0.5% casein solution at pH 9 (using a 50 mM Tris–HCl buffer) to which 10 *μ*l of intestinal enzyme extract was added. This mixture was incubated for 40 min at 17°C. The reaction was stopped by adding 0.5 ml of 20% TCA solution. Blank tubes preparation, spectrophotometry, and the unit of enzyme activity were the same as used at acid proteases. These procedures were similar to those previously performed by Casaretto et al. [71].

A commercially available enhanced *Escherichia coli* phytase (Quantum® Blue from AB Vista, powder) was extracted from their matrix at 0.1% w/v in distilled water by stirring at 4°C for 4 hr. Phytase activity was determined based on Engelen et al. [33]. The mixture consisted of 600 *μ*l of 5.1 mM sodium phytate in acetic buffer (pH 5.5) as substrate and 300 *μ*l of enzyme solution. The incubation was performed for 30 min at 37°C and stopped with ammonium molybdovanadate reagent. Absorbances were read at 415 nm in a spectrophotometer. Phytase units (FTU) were defined as the amount of enzyme that releases 1 *μ*mol of inorganic orthophosphate per minute in the conditions descripted. All measurements were carried out in triplicates.

### 2.4. *In Vitro* Assay

A randomized complete design was carried out, the experimental unit being the proposed bioreactor. The treatments consisted of the combination of five raw materials (*L. mutabilis* D, *L. mutabilis* W, *L. angustifolius* D, *L. angustifolius* W, and SBM) and a single phytase dose (0 FTU/kg DM; 2,500 FTU/kg DM). *In vitro* assays consisted of a two-step digestion, according to Morales and Moyano [37]. The gastric stage in the reaction chamber contained the mixture of 500 mg of each substrate with 12 ml of buffer Gly-HCl and 0.4 ml from stomach enzymatic extract according to the *E* : *S* ratio (Equation (1)). The volume was completed with buffer Gly-HCl to set pH at 2.5, as previously performed by Morales et al. [38], and 250 *μ*l of phytase dose or distilled water to match the final volume of 20 ml. This first incubation was performed for 1 hr with continuous stirring (200 rpm) over a multiple magnetic stirrer (Thermo Fisher Scientific™ Cimarec™ i Poly 15, Waltham, Massachusetts, USA). After 60 min, the pH was raised to 8 with NaOH 1M and buffered with 3 ml Tris–HCl 25 mM. At that time, 0.8 ml of intestinal enzyme extract was added according to the *E* : *S* ratio, and the internal chambers were set. Samples were taken at the beginning (0 min) and at the end (60 min) of the gastric phase. Sampling of the intestinal phase was subdivided into intervals at 0, 30, 60, 120, and 180 min. All trials were performed in triplicate.

(1) $$\begin{array}{c} E:S=\frac{\left(\text{Acid or alkaline protease activity},U/g\right)\times \left(\text{tissue weight},g\right)}{\left(\text{Daily prot}.\text{ intake},gDM/\text{day}\right)\times \left(\text{number of feed rations},{\text{day}}^{-1}\right)}. \end{array}$$

Released soluble phosphorus was measured with molybdovanadate reagent at 415 nm [33]. The total concentration of AAs released as a result of the hydrolysis was measured as m.Eq of tyrosine (Tyr) at 280 nm using 1,280 M^−1^/cm as molar extinction coefficient [39, 40]. The concentration of soluble protein was determined by the Coomassie blue dye method, according to Bradford [41], using 1 mg/ml bovine serum albumin as standard at 595 nm.

### 2.5. Statistical Analysis

The results are presented as means of the total amount of product released over time during the different digestive phases. Results were expressed using arithmetic mean as a measure of central tendency and standard deviation as a measure of dispersion. The data were statistically analyzed by means with one-way ANOVA and two-way ANOVA. Differences between means are significant at *p*  < 0.05. Tukey test was applied to compare means after verifying significant effects. All the analyses were performed using InfoStat® software.

## 3. Results

### 3.1. Raw Materials Characterization

MDS dimension coordinates showed different associations with nutritional variables. Dimension 1 was negatively correlated with the content of CP and positively with the content of CF. On the other hand, dimension 2 showed a negative correlation with ash content. The MDS plot indicates that the dehulling process moves the composition of lupin meals in the same direction, regardless of the species. In general, after removing the hull, lupin kernels presented higher contents of CP, TL, and, to a lesser extent, ash, whereas the content of CF is strongly reduced. Phytate-P was also increased after removing the hull, but ICP-P remained approximately constant. These are general trends, but dehulling did not seem to equally affect all lupin varieties (Figure 1), and the composition of *L. mutabilis* (or SBM) did not change as strongly as those of *L. albus*, or *L. angustifolius*. Another remarkable feature in the MDS plot is the clustering of samples coming from the same species.

### 3.2. Nitrogen and Phosphorus Bioaccessibility

**Figure 2:**
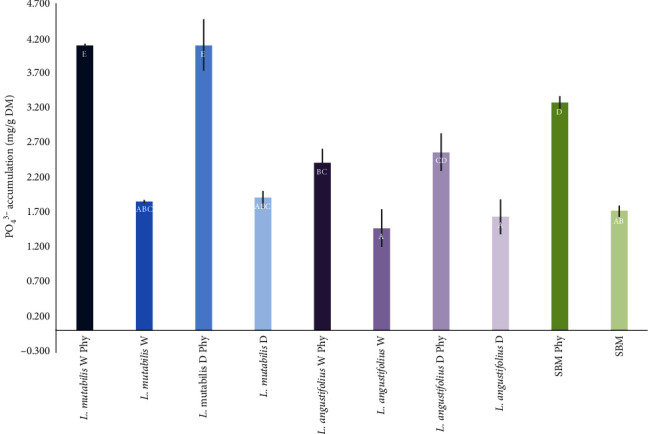
Total accumulated phosphate (mg of PO_4_^3−^ accumulated per mg of DM of substrate) at the final stage (180 min) of the intestinal stage. Mean and SD are presented (*p*-value < 0.0001; *n* = 3; significative differences between treatments indicated in white bold letters, Tukey *∝* = 0.05).

**Figure 3:**
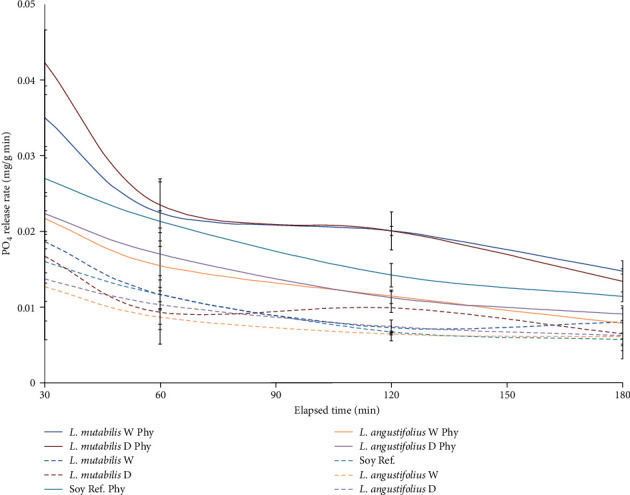
Phosphate release rate (mg of PO_4_^3−^ release per g of DM of substrate per minute) during gastrointestinal simulation. Same color indicates the same species, while full lines stand for variety with phytase dose, and dotted line indicates no phytase treatment. Mean and SD are presented (*n* = 3).

The inclusion of a phytase dose of 2,500 FTU/kg DM increased the levels of PO_4_^3−^ released throughout the intestinal phase by 128.9% ± 8.1% for *L. mutabilis* W, 116.8% ± 16.5% for *L. mutabilis* D, 71.1% ± 13.1% for *L. angustifolius* W, 63.8% ± 12.3% for *L. angustifolius* D, and 87.6% ± 4.7% for SBM compared to controls without phytase (*p*-value < 0.0001). Lupin *L. mutabilis* and SBM registered higher PO_4_^−3^ release than *L. angustifolius* regardless of phytase treatment or seed processing (*p*-value < 0.0001). The variety *L. mutabilis* without shell (*L. mutabilis* D) presented the greatest release of phosphate groups (PO_4_^−3^) at the end of the intestinal phase (4.098 ± 0.021 mg/g), not showing significant differences with respect to the same variety with the hull (4.096 ± 0.374 mg/g) when treated with phytase 2,500 FTU/kg DM. SBM treated with phytase showed a lower response to phytase than *L. mutabilis* (3.272 ± 0.086 mg/g), exceeding the response of *L. angustifolius* (2.398 ± 0.198 and 2.561 ± 0.197 mg/g for *L. angustifolius* W and *L. angustifolius* D, respectively) suggesting that there is an interaction effect between the variety used and the application of this dose of phytase (*p*-value = 0.001). The release of PO_4_^−3^ in *L. mutabilis* D was 25% higher, while *L. angustifolius* D was 22% lower than the reference SBM when treated with phytase 2,500 FTU/kg DM (Figure 2). The soluble P release rates are presented during the intestinal phase, where the highest rates obtained for *L. mutabilis* W, *L. mutabilis* D, and SBM treated with phytase 2,500 FTU/kg DM are observed (Figure 3).

**Figure 4:**
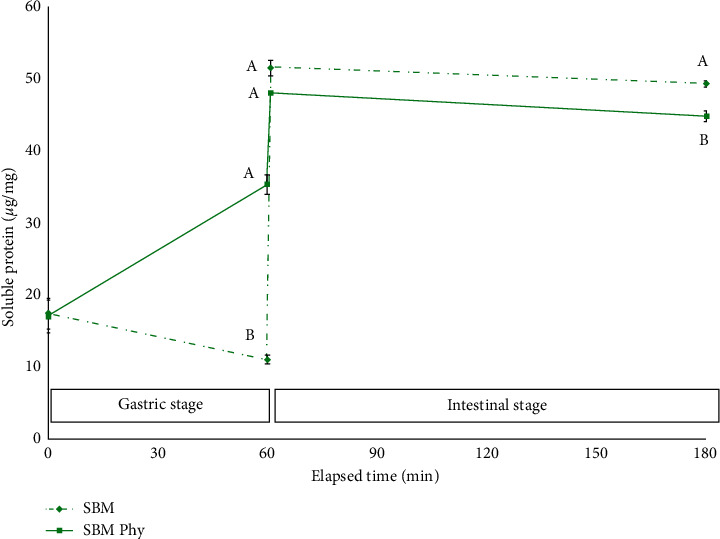
Availability of soluble protein during the gastric and intestinal phase in the digestive simulations of SBM substrate (*µ*g per mg of DM of substrate) for phytase treatment (full line) and control (dotted line). Mean and SD are presented (*n* = 3). Letters in the same color tone indicate differences with Tukey (*∝* = 0.05) for the same time of measure.

**Figure 5:**
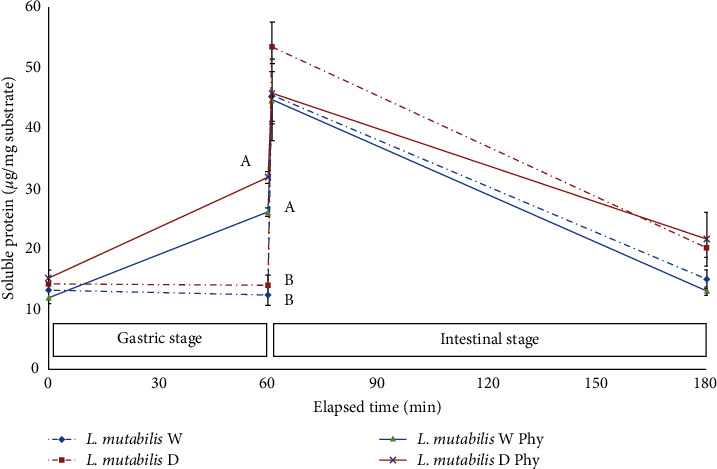
Availability of soluble protein during the gastric and intestinal phase in the digestive simulations of *L. mutabilis* substrate (*µ*g per mg of DM of substrate) for phytase (full lines) dehulled (violet) and control (dotted lines) dehulled (red), phytase whole (green), and control whole (blue) treatments. Mean and SD are presented (*n* = 3). Letters in the same color tone indicate differences with Tukey (*∝* = 0.05) for the same time of measure.

The results of soluble protein during the gastric and intestinal phases (gastric phase represented from 0 to 60 min and intestinal from 60 to 180 min, respectively) were plotted to analyze the impact of phytase, the different varieties, and the presentation of the grain in the case of lupin varieties. The graphic representation of the effect of phytase on the availability of soluble protein during the gastric phase in the digestive simulations of SBM (Figure 4) and lupin *L. mutabilis* (Figure 5) was notorious. Finally, a striking decrease in soluble protein levels during the intestinal phase has been observed in all treatments performed on lupin compared to the levels obtained in SBM.

**Figure 6:**
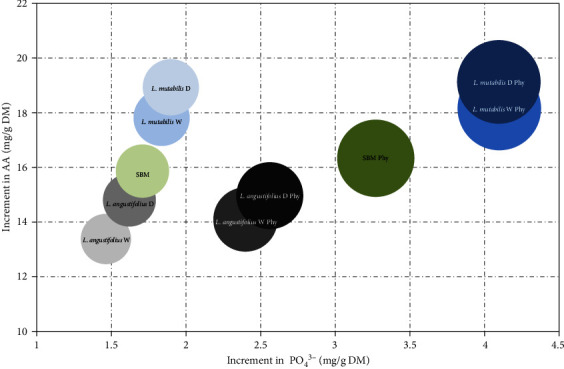
Increment in soluble phosphate (PO_4_^3−^) and amino acids (AAs) bioaccesibility from SBM and assessed lupin meals. Mean and SD are presented (*n* = 3).

**Figure 7:**
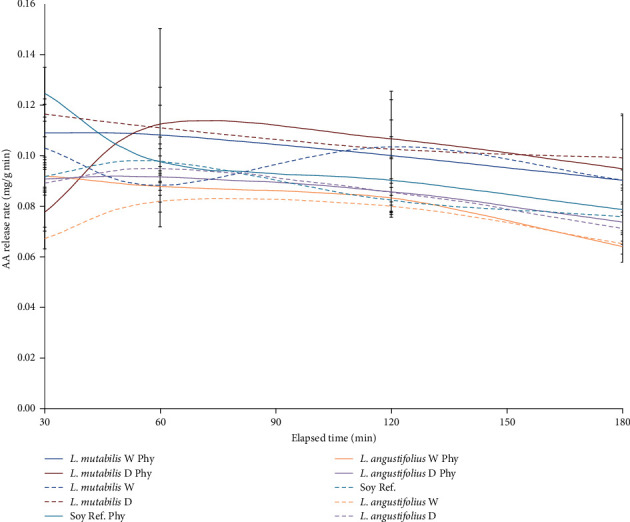
Amino acid release rate (mg released per mg of DM of substrate) during the intestinal stage of gastrointestinal simulation. Same color indicates same variety, while full lines stand for variety with phytase dose, and dotted line indicates no phytase treatment. Mean and SD are presented (*n* = 3).

Regarding the bioaccessibility of AAs in varieties treated with phytase, the increase with respect to the control was 2.3% for *L. mutabilis* W, 1.2% for *L. mutabilis* D, 6.0% for *L. angustifolius* W, 1.4% for *L. angustifolius* D, and 2.0% for SBM. The AA release rates during the intestinal phase were plotted comparatively demonstrating that the effect of phytase is more variable on the release of AAs than documented with the release of phosphates (Figure 6). The variety *L. angustifolius* W accumulated a lower amount of free AAs than SBM and *L. mutabilis* W at the end of the intestinal phase (Figure 7) when phytase was applied (14.978 ± 4.781 mg/g, 16.360 ± 6.244 mg/g, 19.125 ± 3.593 mg/g, respectively), still not resulting in significant differences (*p*-value = 0.8532).

## 4. Discussion

Hulls of different Lupinus species are generally rich in structural polysaccharides and nearly devoid of other macronutrients [42–44]. Accordingly, seed dehulling is expected to promote a decrease in CF and an increase in CP and TL, as observed in this work and previously reported by other authors working with *L. angustifolius* [45]. The increase in phytate-P has also been reported in *L. luteus* dehulled seeds [42], probably indicating that the bulk of phytate of the seed was not distributed near the hull but across the cotyledon mass. This increase in phytate concentration due to hulling supports the convenience of adding phytases in the formulation of the feed when hulled lupine meals are included, as has been tested in the present work. Currently, there are several *in vitro* models to explore the kinetics of nutrients in the digestive tract of fish that can be adapted to different experimental situations [46, 47]. The strength of these methods is the possibility of repeating the experiences in identical conditions and in a serial way [48, 49]. This means that the exploratory possibilities of feed, formulations, and additives can be replicated in a matter of hours or days, allowing for *in vivo* tests to be carried out with a more precise knowledge of the task to be performed and the objectives expected in each case.

The results obtained through the GIM (gastrointestinal *in vitro* model) showed that phytase can substantially improve phosphorus digestibility in *L. mutabilis*, *L. angustifolius*, and SBM. This effect coincides with the results obtained by other authors, who have reported an increase in the bioaccessibility of P due to the effect of phytase both in experimental conditions *in vitro* [37, 38, 49], as well as in *in vivo* experiments [13, 50–54].

According to GIM results on P, the increase in the release of soluble P by phytase activity was higher in *L. mutabilis* when compared to *L. angustifolius*. This effect can be explained by the content of IP6 present in each variety. In the case of variety *L. mutabilis*, with a content of 0.33% of natural phytic acid content, the bioaccessibility of P was increased by phytase by 120% compared to the control without phytase, while the variety *L. angustifolius*, with less than half of native IP6 (0.11%), showed only a 60% increase in the release of P by the effect of said enzyme. This result is in keeping with the kinetic characteristics of wild-type phytases of *E. coli*, like AppA2 phytase, whose Km is close to 135 *µ*M [55]. In comparison, the concentrations of phytate in the GIM were, at the most, 75–80 and 24–27 *µ*M for *L. mutabilis* and *L. angustifolius*, respectively.

This information indicates that depending on the ingredient used to formulate feed for aquaculture, the effect of phytase will be variable and will depend on the amount of native phytic acid in the diet. In this sense, it is important to know not only the IP6 content of each ingredient but also to have information about the response of each ingredient to the inclusion of phytase. Thus, depending on the proportion of ingredients that include a base formula of a particular fish feed, it will be necessary to include a certain quantity of dephosphorylating enzyme in the diet.

Determining the optimal phytase dose of a diet is usually a controversial issue. Previous studies indicate that the optimal dose in a fish diet could be close to 1,000 FTU/kg DM [51, 52, 54, 56–59]. The exhaustive study of each of the ingredients used in the diet, as well as its responses to phytase, becomes essential for an adequate use of this enzyme, contributing to optimize its dephosphorylating action under the conditions of temperature and gastrointestinal environment of the fish and, therefore, reducing the cost of using excessive doses of said enzyme.

In the present study, a positive effect of phytase on the solubility of the protein during the gastric phase has been observed, highlighting the responses of *L. mutabilis* and SBM over those found in *L. angustifolius*. It seems that the highest content of soluble protein in the dehulled *L. mutabilis* and SBM is partially explained by its higher total protein content, and it could be necessary to take this phenomenon into consideration in future experiments.

In experiments carried out by other authors, the presence of dietary IP6 reduced the solubility of casein and SBM proteins during the gastric digestion phase of the fish [38]. On the other hand, several authors found varied mechanisms of interaction between IP6 and dietary proteins depending on the pH gradient of the digestive environment [60–63]. In the stomach, at acidic pH and below the isoelectric points of proteins, binary protein-IP6 complexes are formed by salt bonds with the *α*-NH_2_ terminal group and the *ε*-NH_2_ group of lysine, the histidine imidazole group, and the positively charged guanidine group of arginine [64]. Phytase would be acting during the gastric phase, catalyzing the rupture of these binary and other tertiary insoluble complexes by dephosphorylation of IP6 phosphate groups [65]. As a result, complexed proteins were released and solubilized, as are small peptides, being exposed to gastric hydrolysis. This would explain the higher protein solubility observed during the gastric phase in phytase treatments (2,500 FTU/kg DM) compared to the control treatments (0 FTU/kg DM). On the other hand, no clear effect of phytase on AA release has been observed, and no increase in protein solubility was observed during the intestinal phase. This result coincides with several previous studies that reported the absence of phytase effect under alkaline pH conditions in certain substrates such as soy and lupin [38, 66], while these same authors found a positive effect of phytase under an alkaline environment in other substrates such as peas, chickpeas, and beans.

Regarding the decrease in soluble protein levels during the intestinal phase of lupin digestion compared to that observed in SBM, this would be explained by the de novo formation of binary and tertiary complexes by salt junctions between protein, IP6, and cations by changing the pH of the environment during the transition from gastric digestion to intestinal digestion [49]. The formation of these complexes negatively affects the enzymatic hydrolysis of protein molecules [63].

Considering the results obtained in this work, the use of the enzyme phytase (in this case of bacterial origin) as a feed additive in fish could favor the use of protein and phosphorus present in ingredients of plant origin such as lupin, contributing to a reduction in costs due to the replacement of a scarce input, such as FM, and helping to protect the environment by reducing the amount of P released by the fish. The use of phytase as a feed additive in fish would represent a step towards the efficient utilization of the nutritional virtues of the feed and could help to reverse or stop processes of contamination by excess of unused nutrients that end up accumulating in the environment through the emitted feces for farmed fish.

In this sense, works published by various authors have documented that the dephosphorylation of IP6 by the action of phytases has never been complete, recording average releases of between 40%–60% of the P contained in dietary IP6 [51–53, 67] and in some cases up to 80%–90% [68, 69]. This indicates that there is still much to develop in this regard, considering it interesting to carry out experiences that aim to find the optimal dose or level of phytase inclusion [70] as well as the appropriate technology to include it more efficiently by studying its productive, economic, and environmental impact.

## 5. Conclusions

According to our results, the dehulled *L. mutabilis* lupin has a high total phosphorus content, of which about 50% corresponds to phytic acid or phytate. In formulations, including phytase 2,500 FTU/kg MS, the bioaccessibility of protein and phosphorus was higher than for the rest of the treatments. The development of feed formulations that include a certain amount of dehulled *L. mutabilis* lupin and commercial phytase enzyme may be beneficial for the economic outcome of rainbow trout production. It remains a challenge for the future to determine what quantities of ingredients and additives could maximize the economic and environmental benefit and what is the best technology to incorporate it at an industrial level.

## Figures and Tables

**Table 1:** Proximal composition of different lupin cultivars and soybeans with (whole) and without (dehulled) seed hull.

Grain process	Scientific name	Variety code	Dry matter (%)	Crude protein (%)	Total lipids (%)	Crude fiber (%)	Ash (%)	NFE (%)	Gross energy (MJ/kg)	Phytate P (%)	ICP P (%)
Dehulled	*L. albus*	LU Dic 2011	89.44	44.81	12.37	2.54	3.62	36.65	21.757	0.16	0.37
		Energy 2011	89.46	45.09	12.22	2.69	3.67	36.33	21.879	0.16	0.37
		E12 2011	95.31	45.50	12.11	2.83	3.54	36.01	22.023	0.16	0.39
		E15 2011	92.33	45.52	13.41	3.14	3.57	34.36	22.125	0.15	0.38
		Rumbo 2011	91.35	48.77	10.45	3.14	3.88	33.76	21.305	0.27	0.48
		Pecosa 2011	91.18	46.67	12.83	3.89	3.88	32.71	22.148	0.20	0.42
		Rex 2011	90.24	52.92	10.26	3.95	3.87	29.00	21.644	0.27	0.50
	*L. angustifolius*	Lila 2011	90.31	41.74	8.31	3.62	3.02	43.31	20.893	0.14	0.34
		Walan 2011	94.08	43.71	7.26	3.34	3.18	42.51	20.414	0.13	0.35
		Gungurru 2011	93.80	41.05	7.24	2.92	3.02	45.76	20.423	0.17	0.36
	*L. mutabilis*	E 44	92.47	46.46	19.85	2.95	4.71	26.02	23.721	0.34	0.7
		E 66	92.88	48.17	19.09	5.18	4.54	23.02	23.564	0.31	0.66
	*Gyicine max*	SBM	89.10	46.00	1.68	4.00	6.62	30.80	17.427	0.43	0.68

Whole	*L. albus*	LU Dic 2011	89.29	36.17	10.01	16.81	3.31	33.70	21.045	0.12	0.35
		Energy 2011	88.33	36.07	9.67	16.80	3.32	34.14	21.110	0.14	0.37
		E12 2011	92.40	38.22	10.98	11.59	3.31	35.91	21.363	0.15	0.39
		E15 2011	93.62	38.33	10.79	12.57	3.25	35.06	21.199	0.13	0.40
		Rumbo 2011	91.77	39.98	8.21	14.24	3.56	34.02	20.336	0.20	0.48
		Pecosa 2011	91.58	38.85	11.09	12.39	3.54	34.14	21.281	0.17	0.44
		Rex 2011	90.36	45.67	8.54	12.38	3.63	29.78	20.875	0.24	0.51
	*L. angustifolius*	Lila 2011	92.17	33.24	6.33	18.62	2.84	38.97	19.869	0.11	0.35
		Walan 2011	95.04	33.19	5.93	16.68	2.85	41.35	19.721	0.11	0.35
		Gungurru 2011	91.79	30.62	5.84	17.90	2.81	42.82	19.681	0.12	0.36
	*L. Mutabilis*	E 44	92.43	41.26	17.88	7.90	4.31	28.66	22.996	0.33	0.77
		E 66	88.08	45.54	16.74	8.95	4.37	24.41	23.052	0.27	0.72
	*Gyicine max*	SBM	89.81	44.00	1.39	6.00	6.24	32.18	17.417	0.44	0.69

## Data Availability

Data are available on request by contacting Rosendo L. Azcuy (azcuy@agro.uba.ar).
